# Feasibility and Acceptability of a Tailored Infant Safe Sleep Coaching Intervention for African American Families

**DOI:** 10.3390/ijerph18084133

**Published:** 2021-04-14

**Authors:** Trina C. Salm Ward, Jane McPherson, Steven M. Kogan

**Affiliations:** 1 Helen Bader School of Social Welfare, University of Wisconsin-Milwaukee, Milwaukee, WI 53211, USA; 2 School of Social Work, University of Georgia, Athens, GA 30602, USA; jmcpherson@uga.edu; 3 Department of Human Development and Family Science, University of Georgia, Athens, GA 30602, USA; smkogan@uga.edu

**Keywords:** infant care practices, socio-ecological model, safe sleep campaigns, health promotion, social and cultural determinants, sudden unexpected death in infancy, sudden infant death syndrome, infant mortality prevention, infant sleep practices, theory of planned behavior

## Abstract

Background: Approximately 3600 infants die suddenly and unexpectedly annually in the United States. Research suggests limitations of current behavioral interventions to reduce the risk for sleep-related deaths among African American families living in under-resourced neighborhoods. Guided by the theory of planned behavior and the socio-ecological model, the My Baby’s Sleep (MBS) intervention intends to reduce the risk for sleep-related infant deaths while addressing complex needs of African American families living in under-resourced neighborhoods. Objective: To assess feasibility and acceptability of MBS, a 7-month intervention that includes four home visits and multiple check-ins via phone and text message. Methods: This was a single-arm feasibility and acceptability study with quantitative and qualitive measures. African American families were recruited from community agencies that served an under-resourced metropolitan area. Results: Eight families (eight mothers, nine co-caregivers) completed the intervention. Families reported high acceptability of MBS content, process, and format, as evidenced by qualitative data and mean evaluation scores. Conclusion: MBS is feasible and acceptable among African American families living in under-resourced neighborhoods. These results suggest further investigation of MBS intervention efficacy in a large-scale randomized controlled trial.

## 1. Introduction

Approximately 3600 infants die suddenly and unexpectedly each year in the United States (U.S.) of sleep-related causes such as Sudden Infant Death Syndrome (SIDS), accidental suffocation and strangulation in bed, and undetermined causes [1]. African American infants in the U.S. experience Sudden Unexpected Infant Deaths at twice the rate of white infants (at 186.9 infant deaths per 100,000 live births and 84.9, respectively) [1]. Risk for Sudden Unexpected Infant Death and SIDS has been suggested using, for example, the infection hypothesis [2] and the triple risk hypothesis [3,4], which suggest potential underlying vulnerability that may be exacerbated by modifiable behaviors such as maternal smoking, lack of breast feeding, unsafe sleep surfaces, and prone sleeping. Most sleep-related infant deaths occur in environments that have at least one modifiable behavioral risk factor [5,6,7]. Thus, the main focus of risk reduction continues to be modifying infant sleep practices, such as placing infant supine to sleep on a flat, firm surface designed for an infant, with no loose or soft items in the sleep space, breastfeeding, room-sharing, and avoiding smoke exposure [5,6,7,8]. Despite aggressive public information campaigns recommending practices to reduce the risk for sleep-related deaths [9], epidemiological data indicate that risky sleep practices continue at high rates, especially for African American infants living in neighborhoods with limited resources [10,11,12].

Research has documented limitations to current public health safe sleep messaging for African American parents living in under-resourced neighborhoods in the U.S. [10,11,12,13,14,15,16,17,18,19,20,21,22]. African American caregivers living in under-resourced neighborhoods experience multiple barriers to implementing infant safe sleep recommendations and may adjust their infant sleep practices to accommodate their unique needs and environment [10,11,15,17].

These data underscore the need for ecologically appropriate preventive interventions targeted to this population. Systematic reviews of infant safe sleep interventions, in general, show some degree of success in changing some targeted behaviors [21,23,24]. Few studies, however, report intervention effects with vulnerable or other high-risk African American families [21,24]. Moreover, most interventions include a single educational session, likely an inadequate dose of programming to initiate behavior change with vulnerable families [21,23]. The use of single-session programming is also concerning because research has demonstrated that families’ infant sleep practices change over time, especially when they encounter challenges with adhering to recommendations [15,23,24,25,26,27,28,29]. To help address these challenges, multi-session, tailored interventions are needed that respond to a caregivers’ unique circumstances, concerns, and needs, versus providing a standard, “one-size-fits-all” response that is used broadly across all audiences [15,21,22,23,24,28,30,31].

The purpose of this study was to evaluate feasibility and acceptability of a newly developed intervention with eight African American families living in under-resourced neighborhoods. Our reporting is guided by the Template for Intervention Description and Replication [32] and guidelines for reporting non-randomized pilot and feasibility studies [33].

## 2. Materials and Methods

### 2.1. Study Design

This was a single-arm feasibility and acceptability study consisting of both quantitative and qualitive data collection using surveys and interviews with eight African American families. This study was implemented in preparation for a more costly, large-scale randomized controlled trial to assess the efficacy of the My Baby’s Sleep (MBS) intervention.

### 2.2. Participants

A purposive sample of eight women were recruited using recruitment flyers posted in community settings serving expectant African American mothers with limited incomes. Mothers’ eligibility criteria included being at least 18 years of age, identifying as African American or Black, between six and eight months pregnant, and available to participate over the next seven months. Mothers were excluded if they received formal safe infant sleep programming. Mothers identified one to two co-caregivers (whom they expected to share in caring for the infant). Co-caregiver eligibility criteria included being at least age 18 and available over the next seven months.

### 2.3. My Baby’s Sleep Family Intervention

MBS is a theory-informed intervention aimed at reducing behavioral and environmental risk for sleep-related infant deaths while addressing the complex needs of African American caregivers living in under-resourced neighborhoods. MBS is based on the socio-ecological model and the theory of planned behavior. The socio-ecological model identifies multiple contextual levels that influence and are influenced by an individual’s behavior [34]. Specifically, the socio-ecological model suggests that infant sleep practices are affected by factors at the infant, maternal, family and household, and community–societal levels [19]. The theory of planned behavior has also informed infant sleep practice programs [35]. Specifically, the theory of planned behavior suggests that individuals’ intentions and behaviors are influenced by attitudes toward the behavior, perceived behavioral control/self-efficacy, and subjective norms [35,36]. Informed by these frameworks and research with African American mothers [7,12,13,15,17,18,19,20,21,22,28,37], MBS was designed to increase perceived maternal self-efficacy and control, perceived maternal support and cooperation, and knowledge and attitudes toward safe infant sleep recommendations, which are expected to reduce behavioral and environmental risk factors for sleep-related infant deaths.

MBS consists of four face-to-face, in-home sessions with each family over seven months, beginning in the last trimester of pregnancy and ending when the infant is about four months of age—the highest risk period for sleep-related infant deaths [8] (see Table 1 for MBS overview). Session timing is based on research that has illustrated that despite parents’ positive prenatal intentions, actual practices after the infant comes home may not adhere to recommendations [25,26,27,29,38,39]. The intervention process has staged goals emphasizing engagement and trust-building, followed by skill-building and tailored problem-solving, and concluding with maintenance of behavior change over time. Engagement and trust-building is an initial focus because trust in information sources is a significant factor in parents’ decision-making about infant sleep practices [40,41,42].

Intervention materials were organized into a binder that included:

- A structured workbook;
- An appendix of information sheets explaining and citing research on various topics including supine sleep position, calming a fussy baby, establishing sleep routines, infant sleep patterns, and maternal self-care;
- a local resource guide [43];
- brochures from the National Institute for Child Health and Human Development (NICHD) Safe to Sleep^®^ campaign [9]

The workbook provided structured session content and activities with space for caregivers to write responses. Activities included Safe to Sleep^®^ videos [9] and a card sort activity in which caregivers sorted which recommendations were easier or harder to follow. Activities provided a foundation for caregivers to identify and discuss questions and points of confusion. Coaches used the handouts to further explain topics and concerns identified by caregivers. To facilitate and reinforce recommended sleep practices, participants received educational support tools including a safe sleep infant board book [44,45], folding travel bassinet, and pacifiers [21]. MBS coaches identified as African American women, had undergraduate social work training and experience working with families in under-resourced neighborhoods. Coaches completed 20 h of intervention and safe sleep training [8,9,46] and used an intervention manual to guide sessions.

MBS is designed to meet the needs of African American families with limited resources and introduces five novel perspectives and practices: social network engagement, client-centered approach, tailored information, risk reduction, and home visits.

#### 2.3.1. Social Network Engagement

Without a supportive social context, stressed mothers may have limited capacity to improve the infant sleep environment. Research has demonstrated the significant influence of extended family and social network on infant sleep practices [15,17,19,24,28,31,38,41,47,48]. MBS thus invites mothers to identify “co-caregivers”—individuals who will be helping her care for the infant (e.g., father, extended family member)—and engages co-caregivers to participate in the intervention with the mother.

#### 2.3.2. Client-Centered

MBS stresses the expertise of caregivers in understanding their environment by taking a client-centered approach to shared decision-making [31,49,50]. Rather than being passive recipients of safe sleep messages, caregivers actively collaborate with coaches to develop an infant sleep plan designed to reduce risk and that accommodates their family needs.

#### 2.3.3. Tailored Information

Research has suggested the need for tailored interventions that respond to a caregivers’ unique circumstances, concerns, and needs, especially among families that are living in under-resourced neighborhoods [15,21,22,23,28,30,51]. To tailor their responses to specific family needs, MBS coaches ask families about perceptions and reasons behind infant sleep practices. For example, if a family cites cultural or familial tradition, coaches work with families to honor traditions while also identifying opportunities to reduce risk, or if a family cites crying or infant discomfort as a concern, coaches work with families to expand skills in infant soothing.

#### 2.3.4. Risk Reduction Approach

MBS coaches take a risk reduction approach. Applied to infant sleep practices, this approach acknowledges that parents make decisions about infant sleep practices based on what works within their familial and household context. Risk reduction around decisions to bed-share has been suggested by others [19,30,31,46,50,52,53,54,55]. Consistent with a risk reduction approach, coaches acknowledge the possibility of unplanned bed-sharing occurring [56,57] and plan with families how to reduce potential risk if it occurs [53]. For example, if caregivers suspect they may fall asleep while feeding, they might plan to feed baby on a flat adult bed (versus a chair or sofa, which can increase risk of suffocation [8]), and remove suffocation hazards (such as loose bedding, pillows, etc.) prior to bringing baby into the adult bed.

#### 2.3.5. Home Visits

MBS is delivered within the home because home visits: (a) have demonstrated effectiveness for delivering services to expectant women and mothers of young children [58,59], (b) are particularly important for families with limited incomes who may experience barriers to accessing office-based services, (c) are convenient, as caregivers do not need to arrange for transportation or child care for other children in order to participate, and (d) allow coaches to observe infant sleep environments, better understand specific factors influencing caregivers’ decision-making, and thus provide tailored and concrete recommendations for reducing risks.

### 2.4. Procedures

Interested women contacted the study team via phone call, text, or email. Women were phone-screened for eligibility; eligible mothers were mailed an informed consent, and an enrollment home visit was scheduled. At enrollment, women identified co-caregivers who might participate. The study team assisted with recruiting co-caregivers by providing an introductory letter, informed consent, and follow-up call. Co-caregiver informed consent and baseline were completed at a home visit or via phone. The enrollment visit lasted approximately one hour during which the study was explained, written consent obtained, and baseline data collected.

Coaches completed intervention sessions with mothers and co-caregivers while a research assistant video- or audio-recorded the session. To maintain intervention fidelity, the lead author observed session recordings, provided feedback, and consulted with coaches for future sessions. At the end of each intervention session, participants completed a brief paper-and-pencil survey regarding their experience. Surveys included questions about future target variables, including perceived maternal self-efficacy and control, perceived maternal support and cooperation, infant sleep knowledge and attitudes, and infant sleep intentions and practices. Upon completion of the MBS, mothers and co-caregivers completed a 30 min semi-structured interview about their experiences and satisfaction with MBS and suggestions for improvement. The semi-structured interviews were completed by the PI and research assistants (coaches were not present during the interviews). Mothers received up to $130 in gift cards over the study period, and each co-caregiver could receive up to $50. All subjects gave their informed consent for inclusion before they participated in the study. The study was conducted in accordance with the Declaration of Helsinki, and the protocol was approved by the University of Wisconsin-Milwaukee Institutional Review Board (protocol #18.077) on 3 November 2017.

### 2.5. Measures

#### 2.5.1. Feasibility

To assess feasibility, data were collected on recruitment and retention, intervention delivery, and barriers and facilitators of implementation. Feasibility data were collected using recruitment and visit logs that included visit dates and details about barriers and facilitators to completing on-time visits. Intervention delivery was measured using the visit log and fidelity checklists completed by research assistants while viewing video recordings. Coaches also provided details on barriers and facilitators of implementation. Feasibility of data collection protocols was determined by assessing time spent completing surveys and survey completeness.

#### 2.5.2. Intervention Acceptability

Intervention acceptability was assessed via participant self-report of engagement with coaches and utility of sessions and via open-ended questions. Engagement with coaches was assessed after each intervention session using questions from the Working Alliance Inventory [60,61]. Session utility was measured via statements using a 4-point Likert-type scale ranging from 0 (Not true) to 3 (Very true), including, “Today’s visit was helpful”, “I would recommend this visit to my friends”, and “I learned a lot from this visit”. Open-ended questions included: “What did you like most about this visit?”, “What didn’t you like about this visit?”, and “What else would you like us to know?” At the post-intervention visit, the survey included the above statements regarding the overall intervention and open-ended questions: “Would you recommend this program to your friends?”, “In this program, what was the most helpful thing we did?”, “What was the least helpful thing we did?”, “What would you have liked more information about?”, and “What suggestions would you give for a future program?” [62].

### 2.6. Data Analysis

Analyses focused on feasibility and acceptability. Quantitative survey data were analyzed using descriptive statistics. Qualitative data, including written and verbal responses, observational summaries, and coach feedback were organized through thematic content analysis.

## 3. Results

### 3.1. Participants

**Figure 1:**
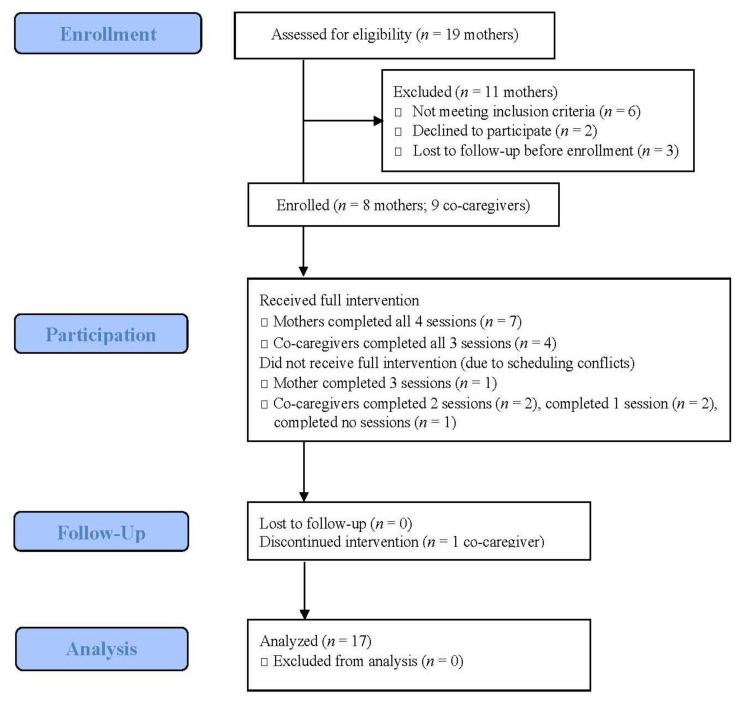
CONSORT 2010 Flow Diagram, modified for non-randomized trial design.

Seventeen participants were enrolled—eight mothers and nine co-caregivers (see Figure 1 participant flow diagram). Mothers were primarily in their mid-twenties, with at least a high school education and living with a partner (see Table 2). The majority of mothers were enrolled in Medicaid, the state health insurance plan, and/or enrolled in the Women, Infants, and Children Supplemental Nutrition Program, indicating that they had limited incomes. Co-caregivers identified as African American and included six partners (infants’ fathers), two mothers (grandmothers to the infant), and a sister (aunt), with an average age of 31.1 (Range: 18–58); half had some high school education.

### 3.2. Feasibility

All but one mother completed all intervention visits; no mothers dropped out or withdrew after study enrollment (Figure 1). One mother was briefly lost to follow-up due to a household move and thus missed one session. Of the nine enrolled co-caregivers, four completed all co-caregiver visits, the remainder completed at least one co-caregiver visit. Co-caregiver drop-out occurred when the primary relationship changed (i.e., couple no longer together) or when employment schedules conflicted with visits. One mother was unable to recruit a co-caregiver due to scheduling constraints.

Almost half (46.9%) of intervention visits occurred outside of the suggested timeframe due to family scheduling difficulties (e.g., work schedules (five families)), infant born early (between 12 and 25 days early) which prevented the second visit from occurring prior to infant’s birth (four families) and limited coach availability (two families). The majority of intervention components were delivered as planned, except for the following three circumstances:

1. One session was shortened for a mother experiencing a family emergency;
2. Content from two sessions was combined when a session was missed due to mother’s household move;
3. Several sessions were modified because a co-caregiver was not present.

Barriers to implementation included slower than expected recruitment—the feedback from community partners was that many families were managing multiple challenges (such as housing difficulties, unemployment, or difficult work schedules) which limited ability to participate.

Facilitators to implementation included:

- Enthusiastic buy-in and assistance from community partners, for example, adding recruitment flyers to prenatal care “welcome” packets and placing flyers in the waiting area;
- Parental interest in participating, including strong engagement with coaches—for example, many families texted the coach within a few days of the infant’s birth, and three mothers referred friends to the study;
- Study cellphones with text messaging capability facilitated communication (most participants preferred text messaging over phone calls) and provided mobile hotspots to play videos at home visits;
- Offering weekend and evening visits allowed more scheduling flexibility, especially for co-caregivers.

Data collection protocols were determined to be feasible—participants completed surveys in about 5–10 min, and most surveys had no missing data. One mother expressed dislike for the repetitive nature of survey questions, and one co-caregiver had difficulty reading but declined to have staff read the survey questions out loud.

### 3.3. Acceptability

Mothers rated most items on session utility as “true” or “very true”, and most coach engagement items as “very true” (see Table 3). Written responses to the question, “What did you like the most about this visit?” included comments such as: “My coaches are friendly and make me feel comfortable”, “The workers are really knowledgeable and supportive”, and “They (coaches) are very helpful and answered all of my questions.” Written responses to the question, “What didn’t you like?” tended to be positive, such as: “There was nothing I didn’t like”. One mother expressed frustration with repetitiveness of content in Session 2 with co-caregivers, noting: “I had to go over things I’ve already done”, while another mother felt the visit was too short and another “wished there was more information”.

Among co-caregivers, most session utility items were rated as true or very true, as were most items regarding coach engagement (see Table 4). Written responses from co-caregivers to the question, “What did you like most?” included comments such as: “How she made things understandable. How they talk and smile” and “I liked everything about the visit but I like the communication the most and that I learned never to put baby in (adult) bed”. Written responses by co-caregivers to the question, “What didn’t you like?” also tended to be positive, for example: “there was nothing bad about it”. One co-caregiver did note that they disliked the video camera.

#### 3.3.1. Positive Aspects of MBS

At the post-intervention visits, when asked if they would refer MBS to a friend, all families agreed. Three mothers further reported that they did refer a friend to the study, and one mother shared MBS materials with a friend. When asked what was most helpful, three mothers noted “everything.” A summary of positive aspects of the intervention is provided below; the number of families mentioning the aspect is listed in parentheses:

- Safe sleep information and resources provided (eight families). Families noted the safe sleep information was helpful, including coaches discussing the reasoning behind recommendations. One mother noted, “just the constant reminder of why it’s important to put the baby on a flat surface”. Among families who had older children, several commented MBS was a helpful reminder, especially as some recommendations had changed over time: “no matter how close your kids are (in age), you always lose some type of information.” A few participants also reported using riskier practices with previous children (i.e., stomach sleeping, bed-sharing) that they did not want to repeat with their new infant. Families reported coaches connected them with needed resources: “She helped me with different resources as far as living-wise. She helped me find a house with 2-1-1 (an information and referral line)”. Families also noted the videos and interactive card sort exercise were useful in processing and summarizing the recommendations;
- Binder/workbook (eight families). Although only about half of the families wrote on workbook pages during sessions, all reported referring back to the workbook and informational handouts between sessions. In particular, participants reported referring to handouts on infant sleep patterns, calming a fussy baby, and maternal self-care. One mother noted: “that’s when I had a hard time, when they are fussy. Like how can I calm them down? In the book it got a lot of options when you can calm them down.” Others noted the local resource booklet [43] and Safe to Sleep^®^ grandparent brochure [9] were helpful;
- Educational support tools (eight families). All families commented positively on the educational support tools. All but one mother reported using the travel bassinet for infant sleep at least once per week. Three mothers used the travel bassinet as an alternative to bed-sharing, and several mothers used it when visiting other homes. All eight mothers reported the safe sleep board book [44] was helpful in reminding them of safe sleep recommendations; seven read the board book to their baby at least once. Two families noted an older sibling “read” the board book to the infant. Four families reported the pacifiers were useful; others did not use them;
- Timing of visits (eight families). All families commented positively on having visits during pregnancy to help with planning for the new baby, for example, “I was nervous, because I didn’t know what to do when she get here”. Several noted visits during pregnancy were helpful to get on the “same page” with co-caregivers: “You helped us prepare for them...you helped us get together, help us get in the mind frame, like, it’s coming, the stuff that’s needed”. Visits after baby was born were also helpful to address challenges to following recommendations. One mother noted: “When you get ready to prepare for him, you think you’re going to be able to follow the steps and have baby the safe way. Then when you have him, you actually see what it’s like”;
- Home visits (eight families). Families noted that home visits were helpful for several reasons, including avoiding transportation and childcare issues, not wanting to take a young infant outside of the house, and the inconvenience of trying to ready an infant and other children for travelling;
- Co-caregiver involvement (five families). Families noted that sharing information with co-caregivers was helpful, as well as helping mother think about who will be involved with infant care. One mother noted: “She (infant’s grandmother) said, ‘oh, I like that. I never had this before’. I think that was very helpful to give the co-caregivers (information), so the mother don’t feel so overwhelmed, like she’s the only one that’s getting all the information when it’s obviously going to be other people watching the baby, too”.

#### 3.3.2. Suggestions for Improvement

Participants and coaches also provided suggestions for programmatic improvement. For example, after completing a few 3rd and 4th intervention sessions, coaches suggested providing anticipatory guidance on infant sleep patterns, addressing “days and nights mixed up”, maximizing maternal sleep, and finding childcare. At the post-intervention interview, families suggested the following:

- More interactive activities (five families). Families suggested adding more interactive activities such as the interactive card sort activity. One father suggested “doing a little (crib) display setup, (asking) ‘how would you set this up?’ seeing how people would set it up...just see where their head is at”. His partner added, “because the doctor, they don’t really show you...but having someone to come in and actually show you how...that would be really helpful”. Another mother suggested physical demonstration could better illustrate suffocation hazards, “show them the dangers of like having the blanket and what that stuff can do or having toys in there”.
- Offering televisits (five families). Several families suggested video calls to add even more scheduling flexibility and to engage co-caregivers with limited availability. Families suggested having some in-person visits (for example, having the first visit in-person and then offering video calls for later sessions (while still offering in-person visits for those who preferred that option).
- Additional content suggestions (three families). Families suggested more detailed content on the following topics: tummy time while awake, specific suggestions for getting baby to sleep when not in physical contact with mother, and pointers for transitioning infants who prefer to sleep on their stomachs away from stomach-sleeping.
- Actively involve siblings (two families). Families also noted that often older siblings are involved in infant care and suggested engaging them in the intervention, for example, “keep the kids involved, the siblings...we say go get the bottle, go grab his diaper...we try to give everybody roles”. Interactive activities such as the crib display could be geared towards siblings “to teach them don’t shake the crib, don’t put toys in there with the baby, don’t put blankets on them”.

## 4. Discussion

Feasibility and acceptability data provided useful suggestions for improving the intervention content and structure for future studies. The intervention structure supported successful coach engagement with families. Post-intervention interviews with families identified several helpful aspects of the intervention, including the safe sleep information and referrals to resources; materials such as workbook, brochures, handouts, and local resource guide; timing and location of visits; and involvement of co-caregivers. Suggestions for improvement included incorporating more interactive activities, offering televisits for follow-up, actively involving siblings in the intervention, and suggestions for additional topics to include (tummy time and sleep location and position pointers). When considering the option of televisits, intervention planners should consider how to manage busy home environments and minimize distraction (for example, at most home visits, other children and adults were present in the home while conducting visits). Refinement of educational handouts, including illustrations, along with hands-on demonstrations of the risks of sleeping prone and on adult beds, may help to increase understanding of safe sleep recommendations. Providing pointers on how to manage when an infant “doesn’t like” sleeping on his or her back or sleeping alone in a crib may help increase caregivers’ options when managing these infant sleep difficulties. Given some of the challenges of completing visits within the desired timeframe, recruiting mothers earlier in the pregnancy could increase the probability of successful completion of two visits during pregnancy and to allow additional time to recruit co-caregivers.

Of important note is consideration that most sudden and unexpected sleep-related infant deaths occur in families experiencing multiple levels of disadvantage, for example, at the individual, family, neighborhood, and societal levels. Specifically, racial disparities in U.S. infant mortality rates have been described within the historical context of race, which incorporates the impact that racism has had on African Americans in the U.S., for example, significantly delayed access to the historical accumulation of wealth and privilege through denial of voting and land ownership rights, as well as structural and personally mediated aspects of racism that continue to impact African Americans today [19,63]. Thus, interventions such as this one are best implemented as part of a multi-level intervention aimed at addressing not only individual family practices, but also neighborhood and societal structures and policies that negatively impact African American families [19,21,24]. While our intervention did not specifically address experiences of structural and personally mediated racism, we did attempt to address the multiple challenges identified by our families through resources and referrals. For example, several enrolled families reported experiencing housing challenges during the study, including housing in disrepair (such as broken plumbing, windows), unresponsive landlords, lack of affordable housing, and eviction. This finding reinforced the necessity of providing a broad array of resources to families living in under-resourced neighborhoods and emphasized the need for tailored interventions that can help respond to a caregiver’s highest priorities and unique circumstances, concerns, and needs [15,21,22,23,24,28,30,31,51].

Study findings have provided several suggestions to refine and improve the MBS intervention. Findings also suggest investigation of the efficacy of the refined MBS intervention in a larger sample using a rigorous randomized controlled trial design that includes specific measurement of the targeted variables of maternal self-efficacy and control, support and cooperation, and knowledge and attitudes toward safe infant sleep recommendations, assessment of behavioral and environmental risk factors in infant sleep environments, and intervention cost. An additional area for future exploration could be formatting the MBS intervention to work as a module that can be incorporated into existing home visiting programs.

### Limitations

Previous studies have noted limitations of self-reported infant sleep practices, especially given the stigma associated with some practices [21,24]. Despite our efforts to engage families in a non-judgmental and non-stigmatizing manner, families may still have reported socially desirable infant sleep practices versus actual practices. Pairing self-report data with observational data of the sleep environment may increase accuracy. Future efficacy studies should also control for possible confounding variables such as parity, infant feeding type, and provider advice. Recruitment challenges were a limitation in this study. The greatest challenge was reaching mothers early enough in pregnancy to enroll and complete visits before the baby was born. Active recruitment methods are recommended, for example, placing a research team member at agencies to facilitate immediate screening and scheduling of enrollment visits, engaging with agencies to assist in actively recruiting eligible mothers, or using social media to aid in recruitment. Our study incentivized data collection and intervention visits which positively impacted retention rates but may limit feasibility in clinical settings. The limited number of subjects, along with the tailoring nature of the intervention, may pose challenges for future investigations of this intervention. Future studies should implement fidelity checks to help address this potential challenge. This feasibility and acceptability study did not recruit controls—it may be helpful to recruit controls in future feasibility studies to assess feelings about randomization, treatment preferences, potential for drop-out and to determine best methods for data collection. Finally, we utilized purposive sampling—recruiting from community agencies that served our target population of African American women with limited incomes—which may have led to selection bias, limiting the generalizability of the study findings. Despite these limitations, this study adds to the limited literature on safe sleep interventions specifically targeting African American families living in under-resourced neighborhoods [21,24].

## 5. Conclusions

My Baby’s Sleep is a promising new theory-guided intervention delivered during the perinatal period that aims to reduce behavioral and environmental risk for sleep-related infant deaths while addressing the complex needs of African American infant caregivers living in under-resourced neighborhoods. Future recommendations are to modify the intervention based upon the available data and test its efficacy in a larger sample. The intervention’s focus on engagement and a tailored approach to addressing infant sleep challenges holds potential for supporting decision-making around safe infant sleep practices for African American families living in under-resourced neighborhoods. Findings suggest suitability for evaluation in a large-scale randomized efficacy trial.

## Figures and Tables

**Table 1:** My Baby’s Sleep intervention overview.

Session Number/Timing	1	2	3	4
	7–8th Month of Pregnancy	8–9th Month of Pregnancy	2–4 Weeks of Age	2–3 Months of Age
**Format**	Mother only	Mother + Co-Caregivers	Mother + Co-Caregivers	Mother + Co-Caregivers
**Session Focus**	Engagement Assessment Goal setting	Engagement Assessment Goal setting	Assessment of sleep safety Skill-building Consensus-building Problem-solving Anticipatory guidance	
**Session Activities**	My goals and hopes for baby My worries and concerns My support system Planning baby’s sleep Handling stress	Our new arrival Supporting mom Baby sleep recommendations How we put it all together	Our baby sleep challenges Making realistic plans together Supporting mom and baby Common sleep challenges Baby sleep patterns Sleep tips for mom	

**Table 2:** Demographics of study sample (*n* = 17).

Characteristic		Number	Percent
**Maternal Demographics (*n* = 8)**			
Age (average, in years)		26.1 (18–32)	
Race	African American	7	87.5
	Bi-racial (African American and White)	1	12.5
Ethnicity	Not Hispanic	7	87.5
	Hispanic	1	12.5
Education	Some high school	2	25
	High school grad/GED	3	37.5
	Some college/technical/vocational	2	25
	Technical/vocational graduate	0	0
	4-year college graduate	1	12.5
Marital status	Living with partner/married	5	62.5
	Single	3	37.5
Insurance type	Private, employer	1	12.5
	Income-contingent state health plan	4	50
	Medicaid	3	37.5
**Co-Caregiver Demographics (*n* = 9)**			
Relationship to infant	Father	6	66.7
	Grandmother	2	22.2
	Aunt	1	11.1
Age (average, in years)		31.1 (18–58)	
Race	African American	8	88.9
	Bi-racial (not specified)	1	11.1
Ethnicity	Not Hispanic	7	77.8
	Hispanic	2	22.2
Education	Some high school	5	55.6
	High school grad/GED	2	22.2
	Some college/technical/vocational	1	11.1
	Technical/vocational graduate	1	11.1

**Table 3:** Mothers’ responses to session helpfulness and coach engagement items.

Item ^1^	Session 1 (*n* = 8)		Session 2 (*n* = 8)		Session 3 (*n* = 7)		Session 4 (*n* = 8)	
	Mean	SD	Mean	SD	Mean	SD	Mean	SD
**Intervention Helpfulness**								
Today’s visit was helpful.	3.0	0	2.9	0.4	2.9	0.4	3.0	0
I would recommend this visit to my friends.	2.9	0.4	3.0	0	2.9	0.4	3.0	0
I learned a lot from this visit.	2.9	0.4	2.9	0.4	2.7	0.5	2.9	0.4
**Coach Engagement**								
I liked my coach.	3.0	0	3.0	0	3.0	0	3.0	0
My coach was helpful to me.	3.0	0	3.0	0	3.0	0	3.0	0
My coach cares about me as a person.	3.0	0	3.0	0	3.0	0	3.0	0
My coach respects me.	3.0	0	3.0	0	3.0	0	3.0	0
My coach listens to me.	3.0	0	3.0	0	3.0	0	3.0	0
My coach had useful recommendations.	3.0	0	3.0	0	3.0	0	3.0	0
My coach understands me and my life.	3.0	0	3.0	0	2.9	0.4	3.0	0

^1^ Items rated on a scale of 0 (Not true) to 3 (Very true).

**Table 4:** Co-caregivers’ responses to session helpfulness and coach engagement items ^1^.

Item ^2^	Session 2 (*n* = 7)		Session 3 (*n* = 5)		Session 4 (*n* = 5)	
	Mean	SD	Mean	SD	Mean	SD
**Intervention Helpfulness**						
Today’s visit was helpful.	3.0	0	3.0	0	2.8	0.5
I would recommend this visit to my friends.	3.0	0	3.0	0	2.8	0.5
I learned a lot from this visit.	2.9	0.4	2.8	0.5	2.8	0.5
**Coach Engagement**						
I liked my coach.	2.7	0.5	3.0	0	2.8	0.5
My coach was helpful to me.	3.0	0	3.0	0	2.8	0.5
My coach cares about me as a person.	2.7	0.5	2.8	0.5	2.6	0.6
My coach respects me.	3.0	0	3.0	0	3.0	0
My coach listens to me.	3.0	0	3.0	0	3.0	0
My coach had useful recommendations.	2.9	0.4	3.0	0	2.8	0.5
My coach understands me and my life.	2.7	0.5	2.8	0.5	2.6	0.6

^1^ Co-caregivers did not attend Intervention Session 1; ^2^ Items rated on a scale of 0 (Not true) to 3 (Very true).

## Data Availability

The datasets generated for this study are available on request to the corresponding author.
